# Maternal mental health and children’s development: a bi-directional relationship?

**DOI:** 10.23889/ijpds.v7i3.1936

**Published:** 2022-08-25

**Authors:** Emily Lowthian, Stuart Bedston, Ashley Akbari, Alan Katz, Katy Huxley, Rhodri Johnson, Sara Madeleine Kristensen, Rhiannon Owen, Chris Taylor, Lucy Griffiths

**Affiliations:** 1 School of Education, Swansea University; 2 Health Data Research UK - Wales and Northern Ireland, Swansea University; 3 Swansea University; 5 Manitoba Centre for Health Policy; 5 Manitoba Centre for Health Policy

## Objectives

Research on bi-directional associations between self-reported caregiver mental health and child development is mixed. Through linkage of a cohort study and primary care data, we examine whether maternal mental health diagnoses, treatment and symptoms are bi-directionally associated with child development, namely emotional and conduct problems, hyperactivity and peer problems.

## Approach

We accessed 14 years of data by linking the Millennium Cohort Study (in Wales) to anonymised individual-level population-scale health and administrative data within the Secure Anonymised Information Linkage (SAIL) Databank. We identified maternal mental health problems using John et al’s (2016) existing algorithm for anxiety and depression diagnoses, symptoms and treatment. We measured child development using parent-reports of the Strengths and Difficulties questionnaire. Outcomes were tested when the child was 3, 5, 7, 11 and 14 years of age. We used Bayesian Structural Equation Modelling, specifically Random-Intercept Cross-Lagged Panel Models, to analyse within and between-person associations.

## Results

We found that mother’s mental health was strongly associated over time, as were children’s development difficulties. Cross-lagged associations from mother to child were weakly positively associated at age 3 to 5 for child total scores, and at age 11 to 14 for child emotional problems. In contrast, child development associations with maternal mental health were significant from age 7 to 11 for total scores, emotional and peer problems, but weakly associated at age 3 to 5, and 11 to 14 for conduct problems. Hyperactivity had few associations. Few associations at the same-time point were found, but emotional problems at age 11 and 14 were positively associated, as were hyperactivity at age 14, and peer problems at age 11. Between-person effects were consistently strongly associated.

## Conclusion

We find mixed evidence for bi-directional associations, but strong between-person associations. Overall development and emotional problem models showed more bi-directional relationships; child development was positively associated with mother’s mental health event at age 7 for all models except hyperactivity, conduct problems were weakly associated at age 5 and 11.

